# Editorial: Real-world evidence and its impact on sustainable health financing, economics and outcomes

**DOI:** 10.3389/fpubh.2026.1906176

**Published:** 2026-07-08

**Authors:** Yu-Hsiang Kao, Xiuli Wang, Yanfei Zhang

**Affiliations:** 1 Global Access, Value and Economics, Intuitive Surgical Inc., Sunnyvale, CA, United States; 2 Healthcare Evaluation and Organizational Analysis (HEOA) Group, West China School of Public Health and West China Fourth Hospital, Sichuan University, Chengdu, China; 3 Health Promotion and Food Nutrition and Safety Key Laboratory of Sichuan Province, Chengdu, China; 4 GE Healthcare, Shanghai, China

**Keywords:** evidence-based decision-making, health economic and outcome research, health economics, health finance, health outcome, health policy, policy impact evaluation, real-world evidence

## 1 Introduction

Real-World Evidence (RWE) has been widely adopted in post-market evaluation and modern healthcare decision-making to support market access and accelerate health policies (1). RWE has also been highlighted as the second leading trend after artificial intelligence (AI) in the top 10 Health Economics and Outcomes Research (HEOR) Trends Report 2026–2027 as published by ISPOR—The Professional Society for Health Economics and Outcomes Research (2). This recognition demonstrates RWE's growing impact and significance for our society. However, challenges persist in the systematic integration of RWE into policy, health financing, and resource allocation decisions. These hurdles include data quality issues, the need for methodological rigor, and the consistent translation of clinical insights into economic practice. Bridging the gap between theoretical efficacy and real-world economic impact, this Research Topic presents 16 high-impact manuscripts from Europe, China, Taiwan, the Republic of Korea, Thailand, and the Middle East to demonstrate the impact of RWE on health financing, economics, and outcomes across four domains.

## 2 Evaluating advanced technologies and precision medicine

New and advanced medical technologies require rigorous and adaptive health technology assessment (HTA) frameworks. A review article by Zhang T. et al. highlighted the rapidly evolving HTA landscape for robotic-assisted surgery, emphasizing the necessity of RWE in capturing its value and guiding regional market access. The concept was further quantified by Hong et al. through a comprehensive systematic review and meta-analysis of the real-world cost-effectiveness of robotic surgery platforms in the Republic of Korea. Additionally, the financial impact of medical devices hinges on long-term lifecycle costs and durability. Sensi et al. conducted a budget impact analysis demonstrating the substantial downstream savings generated by investing in rechargeable deep brain stimulation (DBS) devices for Parkinson's disease compared to non-rechargeable alternatives. Furthermore, precision medicine requires economic efficiency at the population level to secure sustainable financing. Lu et al. investigated the real-world medical cost spending per life-year, lifetime health impact, and incidence of targeted therapies for renal cell carcinoma in Taiwan, providing critical evidence for national insurance reimbursement decisions. Finally, Kantared and Permsuwan assessed the cost-utility of N-acetyltransferase 2 (NAT2) pharmacogenomic-guided tuberculosis therapy in Thailand, showcasing its potential cost-effectiveness for newly diagnosed patients.

## 3 Quantifying the true ledger of disease burden

Sustainable financing requires an accurate accounting of both direct medical costs and indirect losses to human capital and societal productivity. Based on findings from five European countries, Mendes et al. evaluated the association between resource utilization and the economic weight of respiratory syncytial virus (RSV), suggesting a proactive investment in immunization programs for older adult(s) rather than reacting to seasonal hospital surges. Similarly, through a 3-year retrospective follow-up among the working-age population for low back pain in Poland, Szyszka et al. encouraged a shift in funding toward early interventions for chronic disease management.

## 4 Optimizing policy, procurement, and prescribing

RWE is a vital tool for evaluating the financial efficacy of interventions. Raitner et al. provided a blueprint for managing escalating biologic expenditures under the German health insurance system following the strategic introduction of epoetin alfa biosimilars. In China, Yan et al. investigated the effects of the national volume-based procurement (NVBP) policy in Shanghai, demonstrating how centralized, data-driven economic strategies can successfully drive down inpatient costs for osteoporotic hip fractures while maintaining care standards. Beyond traditional pharmaceuticals, Lynch et al. conducted a systematic review of the international health economics of social prescribing. Their review concluded that non-clinical, community-based interventions could yield highly favorable economic returns by significantly reducing the long-term burden on primary healthcare infrastructure. Shifting to consumer economics, Alzuabi et al. mapped out-of-pocket expenditures on over-the-counter medications in the UAE, delivering critical insights into the consumer-driven financial dynamics.

## 5 Valuing the macro-ecosystem and human capital

RWE could expand health economics beyond the hospital-level data to measure the financial impacts across the broader ecosystem. Yang, Zhang et al. utilized multi-center data from western China to demonstrate the value of early Autism Spectrum Disorder (ASD) screening. In a linked thematic study, Yang, He et al. also established the association between urban planning for ASD and public health economics. To address pandemic resilience, Cha et al. emphasized the financial necessity of maintaining a protected healthcare workforce. The broader effect of the environment on health economics and human capital was also explored. Zhao and Huang revealed cross-sectoral benefits, showing how the sports industry directly drives the financial growth and development in the broader health industry. Finally, Zhang S. et al. demonstrated that physical spaces possess economic value by linking improvements in indoor environmental quality directly to measurable gains in cognitive productivity.

## 6 Conclusion and call to action

The 16 studies in this Research Topic prove that RWE is a critical mechanism for aligning clinical innovation with economic viability. Moreover, RWE empowers decision-makers to allocate resources efficiently and optimize procurement pathways. We invite academic researchers, health economists, healthcare professionals, and industry leaders to continuously engage with the evolving field to help build an equitable, evidence-based, and economically sustainable healthcare system.
